# Susana Vázquez Torres: the power of computational protein design 

**DOI:** 10.2471/BLT.25.030525

**Published:** 2025-05-01

## Abstract

Susana Vázquez Torres talks to Ana Lesher Treviño about AI-guided protein design for antivenoms and her aim to improve access to lifesaving treatments in low-resource settings.

*Q: You just completed your PhD —what was your journey like?*

A**:** I was born in the city of Querétaro in central Mexico. Something that really got me excited about science – especially biology – was that my mom had a store where she sold natural products, like herbs and medicinal remedies. That was a big part of my childhood. I grew up surrounded by herbs and ancestral medicinal knowledge. My oldest sister studied medicine, and she used to work in a neurobiology lab, and I remember the first time I went there and saw the mice, the flasks and all the equipment. I didn’t really understand what it all meant, but I was completely intrigued and excited. Later, I moved to Mexico City to pursue a scientific career. I studied at UNAM, the biggest public university in the country, where I majored in biology and later in biomedical research. The education there was excellent – it was the first big door that opened for me. It gave me a lot of opportunities. Thanks to a scholarship, I pursued a master’s degree in the Netherlands. That experience was transformative. I learned English, I connected with scientists from around the world, and I began carving out my own path – my own scientific identity. In the Netherlands, I became more interested in proteins and discovered the work of David Baker. That led to another incredible opportunity: I was able to move to the University of Washington, where I was eventually offered the chance to do my PhD with him. So for me, it’s been a combination of networking and being in the right places, connecting with a supportive scientific community. Each step opened a new door, and that’s how I’ve found myself moving from one country to another, following this path in science.

*Q: When was it that you got interested in protein design?*

A: I was first introduced to protein design and David Baker’s work during my undergraduate studies in Mexico. At the time, his research felt incredibly distant and almost unattainable. Everyone in the scientific community seemed to agree it was only a matter of time before he won a Nobel Prize. His work sparked my curiosity and planted the seed for what would later become a central part of my scientific path.

“These algorithms are trained on massive datasets, essentially capturing decades of experimental biological knowledge.”

*Q: How has the field of protein design changed with the introduction of deep learning and artificial intelligence (AI)?*

A: Before AI and deep learning became mature enough for protein design, most methods were based on physical and chemical parameters – platforms like Rosetta, for example. These tools used detailed parameters to describe proteins, structures and properties and predict interactions, and while they represented cutting-edge science at the time, the success rate was quite low. Designing a protein that simply folded correctly was a major achievement. The landscape changed dramatically with the arrival of AI. Tools like AlphaFold and other deep learning models began outperforming traditional methods, not by explicitly simulating every physical interaction, but by learning patterns from vast databases of experimentally determined protein structures. These algorithms are trained on massive datasets, essentially capturing decades of experimental biological knowledge. With AI, we can now design proteins with significantly higher success rates, better stability and improved ease of manufacture. What used to take years of iterative design and lab testing can now often be achieved in a matter of months. The speed, efficiency and scale that AI enables has opened the door to tackling far more complex and meaningful biological challenges. It hastruly been a night-and-day transformation.

*Q: What led you to focus your research on antivenoms?*

A: I was fortunate to be in the lab at a time when our protein design methods were advancing rapidly and working reliably. That created a unique opportunity – we could ask ourselves not just how to design proteins, but what to design them for. I wanted to choose an application where the impact could be meaningful and where existing solutions were inadequate. Through my research, I discovered that antivenoms are still produced using century-old methods, often resulting in low-quality products with limited availability, especially in low-income countries. Protein design, with its ability to engineer highly specific and stable molecules, seemed like a promising way to create safer, more effective alternatives. Snakebite envenoming disproportionately affects people in the world’s poorest regions – rural, underserved populations with limited access to effective treatment. As someone from Mexico, a country with one of the highest diversities of venomous snakes, this issue felt very close to home. Coming from a public university background, I’ve always felt a strong sense of responsibility to use science in service of society. This project felt like a chance to not just do exciting research, but to apply it where it could truly make a difference – for my country and for others facing similar challenges.

*Q: How can AI-driven protein design lead to better antivenoms?*

A: One of the main advantages of de novo protein design is the ability to produce proteins with high yield and consistency using microbial expression systems. This is a major improvement over traditional antivenom production, which relies on immunizing large animals like horses or sheep with snake venom. Their immune systems then generate a broad and unpredictable mix of antibodies, which must be harvested, processed and purified – a complex and variable process. In contrast, with AI-driven design, we can computationally create proteins that specifically neutralize venom toxins. These designed proteins can then be expressed in microbes such as *E. coli*, where production is consistent and scalable. Fermentation systems allow for the safe and efficient production of large volumes without the variability or ethical concerns associated with animal-based methods. Another important benefit is safety. Traditional serum-based antivenoms contain not only the neutralizing antibodies but also other animal proteins like albumin, which can trigger adverse immune reactions in humans – sometimes even leading to anaphylaxis or serum sickness. With designed proteins produced in microbes, we can purify only the molecules we want, minimizing the risk of harmful side effects.

*Q: How does your research contribute to treatments for neglected diseases?*

A: I believe de novo protein design has significant potential to improve access to effective treatments at multiple levels. First, from a drug development perspective, these tools open new possibilities for researchers – including those in low-resource settings – to contribute to solving local health challenges, such as parasitic diseases or envenomation. Many of the protein design platforms are publicly available and relatively accessible, which helps democratize knowledge. While some background knowledge is necessary, the fact that these are open-source tools lowers the barrier to entry and enables broader participation in therapeutic innovation. From a manufacturing standpoint, designed proteins offer key advantages. First, they can be produced efficiently in microbial systems using fermentation, making production more scalable and affordable. Second, de novo designed proteins tend to have excellent thermal stability, which means they can remain stable without refrigeration – a huge advantage for distributing antivenoms in remote or low-resource areas where cold chain infrastructure is limited. These characteristics could significantly reduce production costs, an advantage particularly in low-income regions where neglected diseases continue to have a disproportionate impact.

“Many of the protein design platforms are publicly available […], which helps democratize knowledge.”

*Q: What are the next steps to bring them to clinical trials and ultimately to the people who need them?*

A: The next step is to focus on designing a product tailored to a specific region and identifying high-priority areas where clinical trials could potentially take place. To move forward, we would need to develop a cocktail of proteins – multiple designed molecules that can neutralize the most medically relevant components of snake venom. These proteins should ideally be broadly specific, meaning they can neutralize toxins from different snake species. This would help create a more versatile and cost-effective treatment. Our current study serves as a proof of concept, where we demonstrated neutralization of isolated toxins. However, the critical next step is to test whether these protein cocktails can offer protection against whole venom, which reflects real-world envenomation more accurately. Once we have a promising combination, we’ll need to address several questions: How can we manufacture this affordably? Is the product thermostable? What is the optimal dose and cost of production? We’ll also need to work with epidemiologists to design rigorous studies that evaluate clinical efficacy. But before all that, the priority is to define the problem clearly. Snakebite envenomation varies significantly by region, each presenting different epidemiological and venom profiles. Identifying a target region and tailoring the product accordingly is essential for the path forward.

*Q: Have you started exploring partnerships to materialize this innovation?*

A: One of the most encouraging outcomes following the publication of our paper has been the interest it generated – several organizations and individuals have reached out to us. Right now, we’re exploring potential collaborations with philanthropic organizations to help raise the necessary funding to advance the project. Although we’ve demonstrated a proof of concept, there’s still a significant amount of research and development required to turn this into a deployable solution. That includes partnerships with epidemiologists to understand the real-world burden, as well as collaboration with experts in drug development and regulatory processes – people who know how to bring a product from the lab to the market effectively and safely.

*Q: What’s next?*

A: The years I spent doing my PhD research really inspired me to use these powerful tools not just for scientific discovery, but to create something that has real impact on society. I recently joined the Spanish National Cancer Research Centre in Madrid, where I’m broadening my expertise – particularly in immunology and cell biology. I believe the intersection of these fields holds tremendous potential. This is a truly exciting time for science – and especially for protein design. We now have tools that were unimaginable just a few years ago, and I want to explore their full potential.

